# Pollution and Health Risk Evaluation at an Abandoned Industrial Site

**DOI:** 10.3390/toxics14010049

**Published:** 2025-12-31

**Authors:** Qing-Zhao Wang, Yu-Qing Zhang, Lin Wang, Yi-Xin Liang

**Affiliations:** 1 Miami College, Jinming Campus, Henan University, Kaifeng 475004, China; 17603955670@163.com; 2 Faculty of Geographical Science and Engineering, College of Geographical Sciences, Henan University, Zhengzhou 450046, China; q18238794306@163.com; 3 College of Engineering, Zhengzhou Technology and Business University, Zhengzhou 451400, China

**Keywords:** abandoned land, soil, heavy metals, pollution assessment, ecological risk

## Abstract

As China’s industrialization progresses, the transformation of site properties across various regions has become increasingly common. Concurrently, with the relocation and market exit of some enterprises, the land occupied by the original factory sites has been developed for other uses. This study provides a comprehensive evaluation of soil and groundwater contamination levels and the associated ecological and health risks in abandoned industrial lands. The investigation focused on analyzing heavy metal and polycyclic aromatic hydrocarbon (PAH) contamination using various assessment methods, including the single-factor pollution index, Nemerow composite pollution index, and potential ecological risk index. These methods were used to assess the contamination levels of 11 heavy metals in both soil and groundwater. Additionally, health risk assessments for PAHs were conducted using the Incremental Lifetime Cancer Risk (ILCR) and Carcinogenic Risk (CR) models, considering both direct and indirect exposure pathways. The results indicated that the average concentration of each heavy metal in the soil did not exceed the screening thresholds, with all Nemerow index values falling below 1, suggesting that the site is not significantly polluted. Ecological risk assessment further revealed that most heavy metals posed minor risks, while some localized areas showed slight enrichment. Health risk assessments for PAHs indicated that, although the risks for both adults and children were within acceptable limits, the ingestion pathway for children showed a slightly higher risk compared to adults. The groundwater quality met Class IV standards, indicating no significant pollution. These findings provide data support and reference for future land-use planning, environmental management, and remediation strategies for abandoned industrial sites.

## 1. Introduction

With the rapid acceleration of global industrialization, contamination in abandoned industrial sites has become a growing concern, particularly in rapidly developing countries like China. While these sites have contributed significantly to social and economic development, the prolonged industrial activities have led to severe environmental pollution. Abandoned industrial sites typically exhibit complex contamination profiles, which include heavy metals and persistent organic pollutants such as polycyclic aromatic hydrocarbons (PAHs). PAHs, a group of persistent organic pollutants, include individual compounds such as benzo[a]pyrene, dibenz[a,h]anthracene, and anthracene. These compounds differ significantly in their toxicity, with some compounds, like benzo[a]pyrene, being highly carcinogenic, while others have relatively lower toxicity. Thus, a detailed assessment of each individual PAH compound is crucial for a comprehensive understanding of their environmental and health risks.

In China, many soils in abandoned industrial sites have been severely contaminated by heavy metals (such as lead and cadmium) and organic pollutants. Heavy metal pollution is widespread in abandoned industrial areas, particularly in sites related to metal smelting and chemical manufacturing. These pollutants can enter the food chain through soil contamination, leading to long-term health impacts on both humans and wildlife [1]. Arsenic (As) is chemically classified as a metalloid, lying between metals and nonmetals. It exhibits metal-like properties, especially in certain chemical reactions where it behaves similarly to metals. However, arsenic is highly toxic and dense, and prolonged exposure to arsenic in the environment can lead to serious health risks, such as skin diseases, cancer, and organ damage. Therefore, although arsenic is classified as a metalloid from a strictly chemical perspective, it is often treated as one of the “heavy metals” and is commonly discussed alongside other heavy metals such as lead, mercury, and cadmium in public health literature. This classification reflects its significant role in environmental pollution and health risks. Meanwhile, PAHs, as typical persistent organic pollutants, are commonly found in industrial waste. They can spread through soil, air, and water, thus becoming a significant environmental and health threat.

In addition to soil contamination, groundwater in abandoned industrial sites is also often polluted. Groundwater, as a crucial water resource, directly impacts the surrounding ecosystem and human health. Due to the lack of effective wastewater treatment facilities at many abandoned industrial sites, pollutants such as heavy metals and organic substances can easily infiltrate and diffuse into the groundwater system, exacerbating pollution severity. This groundwater contamination not only affects local drinking water safety but may also worsen soil pollution through water–soil interactions.

Although numerous studies have revealed the types and sources of contamination in abandoned industrial sites, current research often focuses on the assessment of individual pollutants. There is a lack of in-depth exploration of the dynamic behavior of pollutants across different environmental media (such as soil, groundwater, and air), including their migration, transformation, and interactions [2]. Existing pollution assessment methods are effective in identifying heavy metal contamination in soil, but there is still a significant gap in understanding how pollutants migrate and transform between soil and groundwater, and their combined effects on the ecosystem and human health. This oversight has limited the effectiveness of current remediation strategies, making it difficult to formulate effective restoration plans that address the interactions between different pollutants.

Recent advancements in integrated risk assessment models, such as those proposed in [3,4], have improved the accuracy of contaminant identification. However, a more comprehensive approach is still needed to account for spatial variations in pollution levels and the long-term impacts of these pollutants on public health and ecological systems. Additionally, while the ecological risks of heavy metals have been extensively studied, the health risk assessment of persistent organic pollutants, such as PAHs, remains insufficient, even though these pollutants are increasingly significant in the context of industrial site pollution.

To assess the contamination levels of soil and groundwater in abandoned industrial sites and their potential risks to the ecological environment and human health, this study provides a comprehensive analysis of soil contamination in abandoned industrial lands, with a focus on the long-term impacts of industrial activities in protected areas. While previous studies have identified pollution sources in these regions, our research integrates geochemical analysis, multivariate data analysis, and spatial distribution analysis to assess the environmental and health risks associated with heavy metals and PAHs. This approach offers a more integrated understanding of the pollution legacy in the region, providing valuable insights for future land management and remediation strategies.

## 2. Materials and Methods

### 2.1. The Study Region

The study area is located in Luyi County, Zhoukou City, Henan Province, China. Situated in eastern Henan near the Henan–Anhui provincial border, Luyi County has a warm temperate, semi-continental climate, with an average annual temperature of 14.4 °C, precipitation of 755.2 mm, and wind speed of 3 m/s, predominantly influenced by northerly and northeasterly winds.

Hydrologically, the region lies within the Huai River Basin, with the Guo River serving as the primary watercourse. Originating from Xukou Village east of the Jialu River in Kaifeng City, the Guo River flows southeast through Kaifeng, Tongxu, Taikang, and Zhecheng before entering Luyi County. It traverses 45 km within the county, merging with the Huiji River at Lianghekou in the northeastern Taiqing Palace Township. Major tributaries include the Huiji River, Wuli River, Baigou River, Dasha River, and Tao River.

Pre-industrial land use consisted of undeveloped land, resulting in minimal anthropogenic impact on soil and groundwater systems. Following industrial operations commencing in 2000, the site was utilized for metal alloy storage, leading to significant environmental contamination dominated by heavy metals (e.g., Pb, Cd, As) and polycyclic aromatic hydrocarbons (PAHs).

### 2.2. Sampling Point Setting and Sample Collection

**Figure 1:**
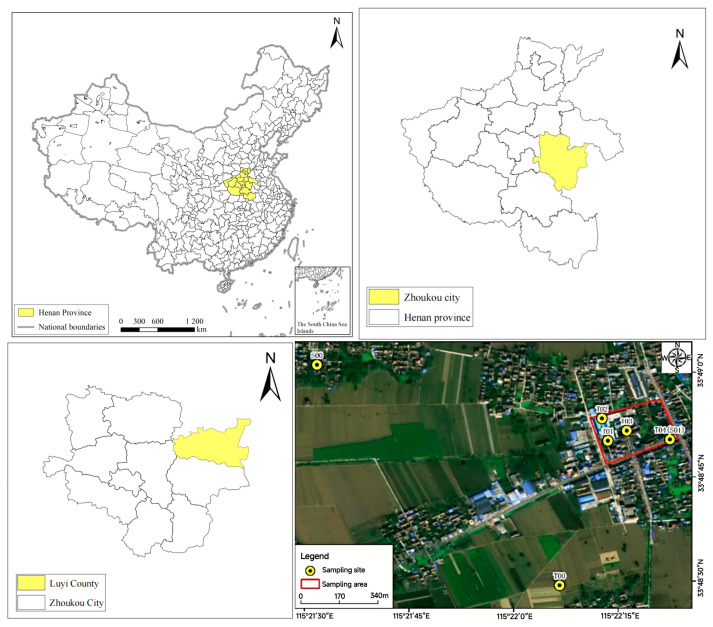
Sampling point location (WGS 1984).

The factory spans an area of approximately 4800 m^2^, with 4 soil monitoring points and 1 groundwater monitoring point. The locations of the sampling points are depicted in Figure 1. Additionally, 1 soil background point (T00) and 1 groundwater background point (S00) were established in the western part of the factory. Soil monitoring point T01 is located in the northern vacant area of the factory, which is a non-production zone. Due to the surrounding buildings and rivers, it was difficult to establish background monitoring samples, so this point was designated as the background monitoring point. Soil monitoring point T02 is situated in the former factory production workshop, identified as the primary suspected contamination area. Soil monitoring point T03 is located in the former production warehouse, which was used for product storage. Soil monitoring point T04 is situated in the southern office area, a major suspected pollution site for production wastewater. Additionally, groundwater monitoring point S01 is also located in this area, as the factory lacked wastewater treatment facilities.

The sampling depths were determined through a comprehensive analysis of pollutant migration, soil stratigraphy, and hydrogeological conditions. Boreholes T01, T02, T03, and T04 were drilled to a depth of 3.0 m, with three discrete samples collected from each borehole (0–0.5 m, 0.5–1.5 m, 1.5–2.5 m), resulting in a total of 12 soil samples. Well S01 (located at the same position as soil point T04) was drilled to a depth of 10 m, targeting the shallow aquifer. A groundwater sample was extracted using the existing on-site shallow well infrastructure. Soil background point T00 consists of surface soil (0–0.5 m) collected from the western perimeter. Groundwater background point S00 was sampled from an existing irrigation well in adjacent farmland to the west of the site, ensuring that baseline hydrological conditions were unaffected by industrial activities. Groundwater was extracted from 0.5 m below the water table.

### 2.3. Heavy Metal Determination

The total analysis of soil heavy metals Cd, Cu, Pb, Ni, Zn, Mn, Co, Se and Be was performed using the HNO_3_-HF-HClO_4_ digestion method, followed by measurement with ICP-MS (X SERIES2, Thermo Fisher Scientific, Waltham, MA, USA). The procedure is as follows: A 0.1000 g sample of air-dried soil, sieved through a 100-mesh, was placed in a polytetrafluoroethylene digestion tube. To the tube, 10 mL of concentrated HNO_3_ was added, and the mixture was shaken and left in a fume hood overnight. The digestion tube was then placed in a digestion system and heated at 120 °C for 1 h. After cooling for 10 min, 3.5 mL of hydrofluoric acid was added, and the mixture was shaken and digested at 140 °C for another hour. After cooling for 10 min, 1 mL of perchloric acid was added, and the mixture was shaken and digested at 160 °C for 1 h, followed by further digestion at 180 °C until no white fumes were observed. After cooling for 30 min, 1 mL of nitric acid solution (V/V 1:1) was added. The solution was then transferred to a 50 mL volumetric flask and diluted to the mark with deionized water. The solution was filtered into a clean white bottle and analyzed using ICP-MS (X SERIES2) [5].

The total analysis of soil As and Hg was performed using aqua regia digestion followed by atomic fluorescence spectrometry (AFS-3100 dual-channel atomic fluorescence spectrometer, Beijing Haiguang Instrument Co., Ltd, Beijing, China). The procedure is as follows: A 0.2000 g sample of air-dried soil, sieved through a 100-mesh, was placed in a 25 mL colorimetric tube. To the tube, 3 mL of aqua regia (V/V 9:1) was added, and the mixture was shaken and placed in a boiling water bath for 1 h, with occasional shaking (2–3 times). After cooling, 5 mL of a mixed solution containing 5% thiourea and 5% ascorbic acid was added. The mixture was shaken well, then diluted to the mark with 3.0 mol/L HCl containing 5.0 g/L tartaric acid. After clarification, the solution was analyzed using atomic fluorescence spectrometry. During the analysis, environmental soil standard sample GSS-8 was used for quality control. The control standard deviation was kept below 5%, and the recoveries of heavy metals ranged between 90% and 110%.

### 2.4. Determination of Polycyclic Aromatic Hydrocarbons

After sample collection, the samples were sealed and stored at 4 °C in a low-temperature, airtight environment [6,7]. Thirteen PAHs were detected, including naphthalene (Nap), acenaphthene (Acy), phenanthrene (Phe), anthracene (Ant), fluoranthene (Fla), pyrene (Pyr), benzo(a)anthracene (Baa), chrysene (Chr), benzo(b)fluoranthene (Bbf), benzo(a)pyrene (Bap), dibenz(a,h)anthracene (Daha), and fluoranthene (Flu). The PAH detection method and quality control followed the USEPA standards [8,9]. The analysis was conducted using a gas chromatography/mass spectrometry system (6890N/5975B, Agilent Technologies, Santa, CA, USA) equipped with an HP-5MS capillary column (30 mm × 0.25 mm × 0.25 μm, Agilent), and the quantification was carried out using an external standard approach based on 13 PAH standard solutions [10]. For each analysis, one blank sample, one parallel sample, and one matrix spike sample were set, and the influence of sample preparation, analysis, and matrix on the results was checked throughout the process using recovery standard indicators. In this study, the sample recovery rate ranged from 95% to 105%, and the relative standard deviation was below 11%.

### 2.5. Soil Pollution Assessment Standards

In the soil pollution assessment, this study combined the Chinese Soil Environmental Quality Standards (GB 15618-2018, GB 36600-2018) with the U.S. EPA Eco-Soil Screening Levels (Eco-SSLs). Given that these two sets of standards had different backgrounds and applicability, they were clearly distinguished as follows:

This study referenced the pollutant concentration limits from the Chinese Soil Environmental Quality Standards (GB 15618-2018) and the Risk Control Standard for Soil Pollution of Development Land (GB 36600-2018) to conduct an initial screening of soil pollution levels. These standards were applicable to soil pollution prevention and ecological protection requirements within China.

For U.S. Standards, additionally, the study referred to the Eco-Soil Screening Levels (Eco-SSLs) published by the U.S. EPA, which were used to assess the potential ecological risks of heavy metal pollution in soil. The application of both standards was clearly distinguished based on their context, ensuring the scientific integrity and consistency of the evaluation framework.

### 2.6. Single-Factor Pollution Index Method

The Single-Factor Pollution Index (*P_i_*) method is used to evaluate the contamination level of individual heavy metal species in soil, allowing for the targeted identification of pollutant-specific risks. This method is widely employed in single-element contamination assessments [11]. The calculation is as follows:

(1) $$P_{i}=\frac{C_{i}}{S_{i}}$$

In Equation (1), *P_i_* represents the pollution index of a specific heavy metal element *i* in the monitored sample, *C_i_* is the detected concentration of element *i* in the soil sample, and *S_i_* refers to the background value or screening value for element *i*. Typically, either the background value of the soil element or the national risk screening value is used as the reference for evaluation. In this study, both the risk screening values from the “Soil Environmental Quality Standard” and the detection values of the monitoring point T00 in the factory area were employed as the standard values [12]. A higher the value of *P_i_* indicates a higher degree of enrichment of the heavy metal in the soil.

Based on the P_i_ value, soil contamination is classified into four levels: when *P_i_* ≤ 1, the soil is considered unpolluted; when 1 < *P_i_* ≤ 2, the soil is considered slightly polluted; when 2< *P_i_* ≤ 3, the soil is considered moderately polluted; and when *P_i_* > 3, the soil is considered severely polluted [13].

### 2.7. Nemerow Comprehensive Pollution Index Method

The Nemerow comprehensive pollution index (P_N_) method accounts for both the average pollution levels of various pollutants in the soil and emphasizes the most severe pollutant’s impact on environment quality [14]. This method provides a more holistic assessment of the pollutant status by considering the overall pollution degree of multiple contaminations, thereby enabling a more comprehensive evaluation of soil pollution. It highlights the harm caused by pollutants and helps gain a deeper understanding of the complexity and severity of pollution. This, in turn, facilitates the development of more targeted and effective pollution control strategies. The calculation formula is as follows:

(2) $$P_{N}=\sqrt{(P_{i\;max}^{2}+P_{i\;ave}^{2})/2}$$

In Equation (2), P_N_ represents the comprehensive pollution index, *P_i__max_* is the maximum single-factor pollution index of each heavy metal in the soil sample, and *P_i__ave_* is the arithmetic mean of the single-factor pollution indices of each heavy metal in the soil sample. According to the Nemerow comprehensive index, the pollution level of heavy metals in soil is classified as follows: when P_N_ ≤ 0.7, the soil is considered to be in a safe condition with extremely low pollution; when 0.7 < P_N_ ≤ 1, it is at a warning level, indicating that close monitoring of soil quality is required; when 1< P_N_ ≤ 2, the soil is slightly polluted, and remediation measures should be taken promptly; when 2 < P_N_ < 3, the soil is moderately polluted, which may significantly impact the ecological environment; and when P_N_ > 3, the soil is severely polluted, necessitating urgent large-scale restoration and remediation efforts to prevent further degradation and ensure the stability and health of the soil ecosystem.

### 2.8. Potential Ecological Risk Index Method

The potential ecological risk index (RI) is designed to quantitatively assess the potential ecological risk of soil heavy metals [15,16]. Its unique advantage lies in its ability to not only assess the impact of individual heavy metal elements on the environment, but also to provide a comprehensive evaluation of the combined effects of multiple heavy metals. As a result, the Potential Ecological Risk Index has become a widely applied and effective method for assessing the ecological risks posed by heavy metals in sediments and soils. This method offers a reliable quantitative foundation for understanding the potential ecological threats posed by soil heavy metal pollution, thereby facilitating more effective environmental research and informing better governance strategies. The calculation formula is as follows:

(3) $$C_{f}^{i}=\frac{C_{0}^{i}}{C_{n}^{i}}$$

(4) $$E_{r}^{i}=T_{r}^{i}\times C_{f}^{i}$$

(5) $$EI=\sum_{i=n}^{n}E_{r}^{i}$$

### 2.9. Pollution Load Index

The pollution load index (PLI) is a method for assessing the comprehensive pollution level of multiple heavy metal elements in the soil of a study area by calculating the toxicity of heavy metal elements [17]. In the formula, PLI represents the pollution load index of the sampling point; C_i_ denotes the measured value, with the unit mg·kg^−1^; B_i_ refers to the soil background value, with the unit mg·kg^−1^; and n indicates the number of elements [18]. PLI ≤ 1 indicates no pollution, 1 < PLI ≤ 2 indicates slight pollution, 2 < PLI ≤ 3 indicates moderate pollution, PLI > 3 indicates severe pollution.

(6) $$PLI=\sqrt[{n}]{\frac{C_{1}}{B_{1}}\times \frac{C_{2}}{B_{2}}\times \cdots \times \frac{C_{n}}{B_{n}}}\;$$

### 2.10. Assessing Health Risks of Soil PAHs

In this study, we applied the Incremental Lifetime Cancer Risk (ILCRs) model to assess the potential health impacts of PAHs in soil on local residents by both adults and children [19,20]. The key parameters in the model were defined and rationalized in detail to ensure the scientific accuracy and reliability of the risk assessment. This study assumed an exposure frequency of 365 days/year for adults (daily exposure) and 250 days/year for children (assuming less frequent exposure). These assumptions were based on the lifestyle and environmental conditions of the local population, reflecting the actual exposure patterns. The exposure duration for adults was set at 30 years, while for children, it was set at 6 years. These assumptions reflect the long-term exposure patterns over the lifespan of local residents, in line with common epidemiological studies. The body weight for adults was set at 70 kg, and for children, it was set at 15 kg. These values were based on the body weight distribution of the local population and were aligned with the World Health Organization’s standards to ensure the parameter settings are reasonable.

The following formulas were used to calculate the intake of PAHs through these three exposure pathways [19]:

(7) $${ILCR}_{cib}=\frac{CS\times {CSF}_{cib}\times \sqrt[{3}]{BW/70}\times {IR}_{cib}\times EF\times ED}{BW\times AT\times 10^{6}}$$

(8) $${ILCR}_{bre}=\frac{CS\times {CSF}_{bre}\times \sqrt[{3}]{BW/70}\times {IR}_{bre}\times EF\times ED}{BW\times AT\times 10^{6}}$$

(9) $${ILCR}_{skin}=\frac{CS\times {CSF}_{skin}\times \sqrt[{3}]{BW/70}\times SA\times AF\times ABS\times EF\times ED}{BW\times AT\times 10^{6}}$$

(10) $$CS=\sum \left({PAH}_{i}\times {TEF}_{i}\right)$$

where *CS* is the concentration of individual PAHs in the soil sample (μg/g); *TEF* is the toxicity coefficient of PAHs relative to benzo[a]pyrene (BaP); *CSF* is the carcinogenic slope factor of PAHs (kg·d)/mg, which is determined based on the carcinogenic potency of BaP. The values for *CSF* ingestion, *CSF* inhalation, and *CSF* skin contact are 7.3, 3.85, and 25 (kg·d)/mg respectively; *CR* the cumulative carcinogenic risk from the three exposure pathways, calculated as follows [19,21]:

(11) $$CR=\sum \left({ILCR}_{cib}+{ILCR}_{bre}+{ILCR}_{skin}\right)$$

For the classification of carcinogenic risk (*CR*), the following thresholds are used: when *CR* < 10^−6^, the risk is considered within an acceptable safety range; 10^−6^ < *CR* < 10^−4^, the risk represents a potential hazard that the human body can tolerate; *CR* > 10^−4^, the risk is considered significant, indicating a potential threat to health [22].

## 3. Results and Discussion

### 3.1. Characteristics of Soil Heavy Metal Pollution and Health Risks

#### 3.1.1. Characteristics of Soil Heavy Metal Pollution

The concentrations of 11 heavy metals (As, Cd, Cu, Pb, Hg, Ni, Zn, Mn, Co, Se, Be) in the soil samples were analyzed, as summarized in Table 1. Their mean levels ranged from 0.052 μg/g for Hg to 513.917 μg/g for Mn. The comparison of the average concentrations to their respective background values revealed varying levels of metal enrichment. For example, the average concentration of Mn was the highest, while Hg had the lowest average concentration.

In terms of the minimum and maximum values, concentrations varied significantly across the samples. The minimum concentrations ranged from 6.970 μg/g for As to 0.004 μg/g for Hg, while the maximum concentrations ranged from 16.800 μg/g for As to 801.000 μg/g for Mn, indicating notable variability in soil metal contamination across the study area. Our research has found that heavy metal pollution mainly originates from historical emissions in abandoned industrial areas, especially the increase in As concentration, which is consistent with the findings of Michels et al. [23].

The standard deviations of these concentrations varied significantly, reflecting the degree of variation in contamination levels. For instance, the standard deviation for Mn was 125.107 μg/g, indicating substantial variability in its concentration, while Hg exhibited a much smaller standard deviation of 0.127 μg/g, suggesting more uniform distribution of this metal in the soil.

To assess the relative variability of the data, the coefficient of variation (CV) was calculated. Hg exhibited the highest CV (2.463), indicating significant variation that may be influenced by anthropogenic activities. Conversely, metals such as Co and Be displayed lower CV values (0.130 and 0.085, respectively), suggesting more stable concentrations of these elements across the study area.

Enrichment factors were calculated to assess the degree of contamination relative to background levels. The enrichment factors ranged from 0.17 for Zn to 1.56 for Mn, with Mn, As, and Hg showing higher enrichment compared to their background values. This suggests that these metals are more concentrated in the soil than naturally occurring levels would suggest. Of particular concern is mercury, with an enrichment factor of 1.57, which could indicate contamination from industrial activities or other anthropogenic sources.

To evaluate the potential ecological risks, Ecological Soil Screening Levels (Eco-SSLs) were compared to the measured concentrations. The Eco-SSLs represent the threshold concentrations above which ecological risks are considered significant. For example, the Eco-SSL for As is 60 μg/g, and for Pb, it is 800 μg/g. The concentrations of As, Cd, and Pb in the studied soil samples were all below their respective Eco-SSLs, indicating that these metals do not pose an immediate ecological threat. However, certain metals such as As, Hg, Mn, Co, Se, and Be exceeded their respective background values, suggesting localized enrichment that could pose potential ecological risks.

The presence of elevated concentrations of Hg, in particular, warrants further attention. Given the high CV for Hg (2.463), it is likely that human activities, such as industrial processes, may have contributed to this contamination.

#### 3.1.2. Single-Factor Pollution Index Method

According to the national standard assessment method in the “Soil Environmental Quality Standards”, the single-factor pollution index (*P_i_*) for all analyzed heavy metals at various monitoring points is below 1, indicating that no pollution occurred according to China’s risk screening thresholds.

Using background values, the single-factor pollution index method was applied to assess soil heavy metal pollution. The analysis of As pollution at the four monitoring points showed that at monitoring point 1, the single-factor pollution index remained below 1, suggesting that the soil at this point is in a good and non-polluted state. However, at the other three monitoring points, the pollution indices ranged between 1 and 2, indicating mild pollution. For Cd, significant enrichment was found only at a depth of 1.5 m at monitoring point 3, below the hardened surface, resulting in a P_i_ value greater than 1, while other monitoring points remained unpolluted. Pb also showed abnormal enrichment at a depth of 0.5 m at monitoring point 2, below the hardened surface, causing the P_i_ value to exceed 1, while other monitoring points showed no pollution. Hg exhibited an exceptionally high concentration at 0.5 m below the hardened surface at monitoring point 4, with a P_i_ value of 13, signifying severe pollution. Other monitoring points had P_i_ values lower than 1 or close to 1, indicating some localized enrichment that requires further monitoring. Cu, Ni, and Zn all showed Pi values below 1, indicating that these metals were unpolluted. Mn, Co, and Be exhibited P_i_ values greater than 1, suggesting mild pollution.

#### 3.1.3. Nemerow Comprehensive Pollution Index Method

By integrating the single-factor pollution index with the Nemerow Comprehensive Pollution Index (NCPI), a dual assessment was performed for 11 heavy metals in 12 soil samples. The NCPI values for all samples were below 1.0, indicating that the overall pollution level of the soil is below the critical threshold for pollution (Table 2).

The soil quality of the study area fully meets the Category 2 land use screening standard as defined by the “Soil Environmental Quality—Risk Control Standard for Contaminated Soil of Construction Land”, confirming that the soil is not significantly polluted and is suitable for industrial or commercial use [18].

A further comprehensive evaluation was conducted by comparing the monitoring area with the background values (T00), as shown in Table 3. The average single-factor pollution index for both the study and background areas was below 1, suggesting that the heavy metal concentrations in both areas are similar. However, the Nemerow comprehensive pollution index values in the study area were generally higher than 0.7, indicating that the overall concentration of heavy metals in the monitoring area is higher than in the background area.

The dual assessment using the single-factor pollution index and the Nemerow comprehensive pollution index confirmed that the soil in the study area is not significantly polluted, with all samples having NCPI values below 1.0. The soil quality meets the regulatory standards for industrial and commercial land use. However, the NCPI values in the monitoring area are slightly higher than those in the background area, suggesting that the heavy metal concentrations in the monitoring area have increased and warrant further monitoring and evaluation to ensure long-term environmental safety.

#### 3.1.4. Potential Ecological Risk Index Method

Based on the national soil heavy metal standards, the potential ecological risk index (RI) method was used to calculate the ecological risk coefficients and risk indices for heavy metals in the soil of the monitoring area (Table 4) [12]. These values help assess the ecological risk level in the study area. The ecological risk index (E^i^_r_) for As, Cd, Cu, Pb, Hg, Ni, Zn, Mn, and Co was calculated. The results showed that the average ecological risk index for all heavy metals was low, indicating that the study area is not severely polluted.

By comparing the ecological risk indices of the monitoring and background areas (Table 5), the results showed that while most heavy metals presented mild ecological risks, Hg and Cd exhibited moderate or higher ecological risks in some samples. Notably, mercury showed a relatively higher risk level in certain samples.

#### 3.1.5. Pollution Load Index

**Figure 2:**
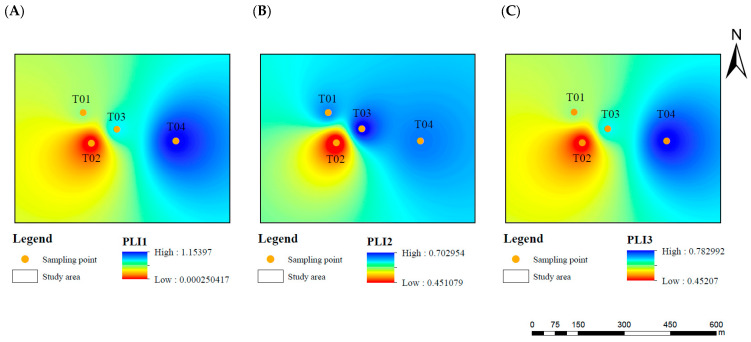
Spatial distribution of pollution load index. (**A**): 0–0.5 m, (**B**): 0.5–1.5 m, (**C**): 1.5–2.5 m.

As shown in Figure 2, the Pollution Load Index (PLI) varies across different soil layers. Only sampling point T041 exhibits slight pollution, primarily due to an abnormal increase in Hg concentration. At all other sampling points, the PLI is below 1, indicating no pollution. The surface layer has a relatively low PLI, and there is a trend of increasing pollution levels with greater soil depth.

#### 3.1.6. Spearman Correlation Analysis

**Figure 3:**
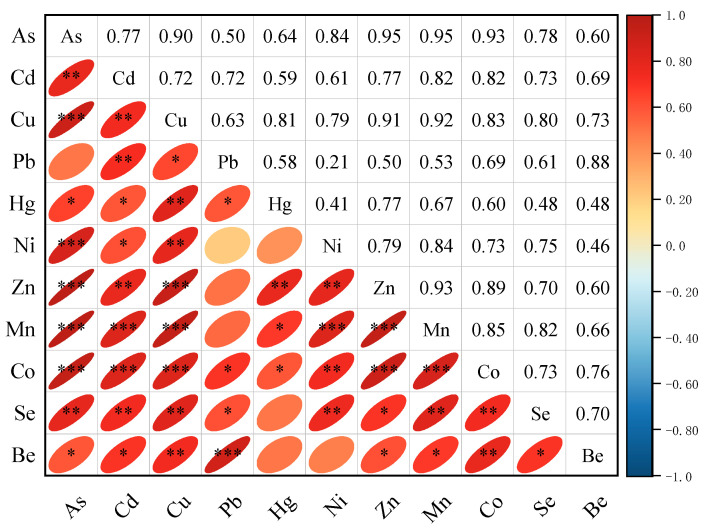
Spearman correlation analysis (* *p* ≤ 0.05, ** *p* ≤ 0.01, *** *p* ≤ 0.001).

After verifying the normality of the data distribution, Spearman correlation analysis was conducted on the heavy metal concentrations, and the results are presented in Figure 3. Generally, the strength of the correlation between two elements reflects their similarity in source, with stronger correlations indicating higher homology. Therefore, correlation analysis can be an effective tool for tracing the sources of heavy metals [24].

Except for Pb, Hg, and Be, all other heavy metals exhibited highly significant positive correlations. Specifically, Pb showed relatively weak correlation coefficients with Ni, Zn, Mn, and As, with values of 0.21, 0.50, 0.53, and 0.50, respectively. Similarly, Be exhibited lower correlations with Hg and Ni, with coefficients of 0.48 and 0.46, respectively. In contrast, Pb and Be demonstrated a highly significant positive correlation, with a coefficient of 0.88. This suggests that Pb and Be may originate from a common source, while the remaining heavy metals likely stem from a different source.

### 3.2. Characteristics of PAH Pollution and Health Risks

#### 3.2.1. Statistical Analysis of PAH Concentration

Table 6 presents the statistical analysis of PAH concentrations in the soil of the study area. The maximum concentrations of all PAHs were below their respective Eco-SSLs (Ecological Soil Screening Levels), indicating that, from an ecological risk perspective, none of the measured concentrations were considered critical. However, the coefficient of variation (CV) for all PAHs exceeded 1, indicating a high degree of variability. This suggests that the concentration of PAHs in the soil is significantly influenced by human activities.

The concentrations of benzo[a]pyrene (Bap) and dibenzo[a,h]anthracene (Daha) were 0.046 mg/kg and 0.118 mg/kg, respectively, both of which exceeded their corresponding Eco-SSLs (1.5 μg/kg and 0.55 μg/kg). This indicated that the concentrations of these two PAH compounds in the soil could potentially pose a threat to the ecological environment. The concentrations of other PAH compounds, such as benzo[a]anthracene and benzo[b]fluoranthene, were all below the Eco-SSLs, suggesting that these compounds did not pose a significant threat to the ecological environment.

Bap is a typical PAH that accumulates in soil and sediments due to its stable structure and environmental persistence. It is classified as a Group 1 carcinogen by the International Agency for Research on Cancer (IARC), with well-established carcinogenic, mutagenic, and teratogenic properties. Bap enters the human body primarily through soil ingestion, with children being more vulnerable due to their frequent hand-to-mouth behaviors. Furthermore, Bap’s hydrophobic and lipophilic properties enable it to adsorb to soil and particulate matter, posing ecological risks by inhibiting microbial activity and disrupting nutrient cycling. Its accumulation in the environment and its potential for biomagnification through the food chain contribute to its status as a key compound for PAH health risk assessments.

Daha is a carcinogenic PAH with significant detrimental effects on both human health and the environment. Prolonged exposure to Daha can increase the risk of skin, lung, and liver cancers due to its ability to bind with DNA and induce mutations. Furthermore, as a persistent organic pollutant, Daha can accumulate in soil, water, and biota, posing a threat to aquatic organisms, plants, and soil microorganisms. It can also act as an endocrine disruptor, affecting the hormone systems of wildlife and humans, leading to reproductive and developmental issues. Given these risks, controlling the environmental concentration of Daha is crucial for the protection of ecosystems and public health.

#### 3.2.2. Health Risk Assessment of Soil PAHs

The potential health risks associated with PAHs in the soil of the study area were assessed using the Incremental Lifetime Cancer Risk (ILCR) and Carcinogenic Risk (CR) models for both adults and children. The assessment considered three primary exposure pathways: ingestion, skin contact, and inhalation. The ILCR and CR values were calculated using the Formulas (7)–(11), and the results are summarized in Table 7.

It was observed that, for both adults and children, the ILCR due to ingestion (among the three exposure pathways) exhibited both average and maximum values greater than 10^−6^, while the ILCR values from skin contact and inhalation were below 10^−6^, remaining within the acceptable safety limits. This suggests that the carcinogenic risks associated with PAHs in the soil of the study area are within the acceptable safety range.

Among the three exposure pathways, ingestion was identified as the primary route of PAH exposure. The ILCR from ingestion accounted for 85.65% (for adults) and 85.71% (for children) of the total carcinogenic risk. Inhalation was the second most significant pathway, contributing 14.9% (for adults) and 14.12% (for children) of the total carcinogenic risk. Skin contact contributed less than 0.01% of the total risk and is negligible in this context.

Furthermore, the calculated CR values indicated that the hazard risk to children’s health was slightly higher than that to adults in this area. Therefore, it is recommended that children be restricted from direct contact with the soil or other contaminated media to minimize potential exposure.

### 3.3. Water Quality and Ecological Risk Assessment

Based on the data presented in Table 8, none of the measured parameters exceeded the Class IV water quality standards outlined in the Reference Groundwater Quality Index (GB/T 14848-2017). Therefore, the groundwater in this study area is not considered polluted.

## 4. Conclusions

This study provides a comprehensive assessment of soil pollution and health risks associated with abandoned industrial lands, with the following key conclusions:

(1) The overall level of heavy metal pollution in the study area is within the non-polluted range, with all soil samples having Nemerow Comprehensive Pollution Index (NCPI) values below 1, indicating that the soil is not significantly polluted. However, localized pollution was observed at certain monitoring points, particularly for As, Cd, and Pb, where the single-factor pollution index ranged between 1 and 2, suggesting mild pollution. At one monitoring point, Hg had a pollution index of 13, indicating severe contamination.
(2) The potential ecological risk index (RI) for soil heavy metals was generally low, with most metals posing a mild ecological risk. However, Hg posed moderate to strong ecological risks at certain locations, highlighting the need for targeted attention in these areas.
(3) The Incremental Lifetime Cancer Risk (ILCR) and Carcinogenic Risk (CR) models indicated that the carcinogenic risks of PAHs in the soil were within acceptable safety limits for both adults and children (CR < 10^−4^). However, the ILCR for children’s ingestion pathway slightly exceeded 10^−6^, suggesting that the health risk for children is slightly higher than for adults. Although the overall environmental quality of the abandoned industrial land meets the regulatory standards for land use, localized heavy metal contamination and the potential health risks of PAHs to children still warrant further monitoring and remediation efforts.

It should be noted that due to site accessibility constraints, the number of sampling points was limited, with the sampling strategy focusing primarily on high-risk areas to provide valuable insights. However, the limited number of sampling points may restrict the generalizability of the results. Therefore, future studies should expand the sampling coverage to enable a more comprehensive assessment, providing accurate data for land-use planning, environmental management, and remediation strategies, thus supporting the safe development and rational utilization of abandoned industrial sites.

## Figures and Tables

**Table 1:** Statistical analysis of heavy metal concentrations in the soil samples.

Heavy Metal	As	Cd	Cu	Pb	Hg	Ni	Zn	Mn	Co	Se	Be
Mean (μg/g)	11.11	0.19	26.92	14.03	0.052	44.33	71.19	513.92	15.96	0.07	2.43
Min (μg/g)	6.970	0.09	14.00	7.70	0.004	32.00	46.20	368.00	11.00	0.03	2.16
Max (μg/g)	16.80	0.46	38.00	29.50	0.45	62.00	86.80	801.00	19.10	0.09	2.79
Standard deviation (μg/g)	2.74	0.10	7.26	6.18	0.13	10.74	13.91	125.11	2.08	0.02	0.21
Limit of detection (μg/g)	0.01	0.01	1.00	0.10	0.002	5.00	1.00	0.10	0.40	0.01	0.03
CV	0.247	0.542	0.270	0.440	2.463	0.242	0.195	0.243	0.130	0.314	0.085
Eco-SSLs (μg/g)	60	65	18,000	800	38	900	2000	2000	70	800	29
Background value (μg/g)	9.00	0.29	33.00	19.70	0.03	108.00	415.00	330.00	14.10	0.29	1.66
TEFs	10	30	5	5	40	5	1	1	5	10	5
Enrichment factor	1.23	0.65	0.82	0.71	1.57	0.41	0.17	1.56	1.13	0.228	1.47

**Table 2:** Single-factor pollution index and Nemerow comprehensive pollution index (P_N_) for heavy metals in the study area.

	As	Cd	Cu	Pb	Hg	Ni	Zn	Mn	Co	Se	Be
Filtering value (μg/g)	60	65	18,000	800	38	900	2000	2000	70	800	29
Pk_i ave_	0.19	0.00	0.00	0.02	0.00	0.05	0.04	0.26	0.23	0.00	0.08
Pk_i max_	0.28	0.01	0.00	0.04	0.01	0.07	0.04	0.40	0.27	0.00	0.10
P_N_	0.24	0.01	0.00	0.03	0.01	0.06	0.04	0.34	0.25	0.00	0.09

**Table 3:** Single-factor pollution index and Nemerow comprehensive pollution index (PN) for heavy metals in the monitoring and background areas.

	As	Cd	Cu	Pb	Hg	Ni	Zn	Mn	Co	Se	Be
T00 (Background value) (μg/g)	9.00	0.29	33.00	19.70	0.03	108.0	415.0	330.0	14.10	0.00	1.66
Pki00_max_	1.00	1.00	1.00	1.00	1.00	1.00	1.00	1.00	1.00	1.00	1.00
Pki00_ave_	0.58	0.73	0.46	0.71	0.89	0.59	0.38	0.66	0.39	0.40	0.57
P_N_00	0.82	0.88	0.78	0.87	0.94	0.82	0.76	0.85	0.76	0.76	0.81

**Table 4:** Potential ecological risk index (standard values) for heavy metals in the soil.

Heavy Metal	Min	Max	Mean	Slight (%)	Moderate (%)	Intense (%)	Very Strong (%)	Extremely Strong (%)
As	7	16.8	11.1	100	0	0	0	0
Cd	0.1	0.5	0.2	100	0	0	0	0
Cu	14	38	26.9	100	0	0	0	0
Pb	7.7	29.5	14	100	0	0	0	0
Hg	0	0.5	0.1	100	0	0	0	0
Ni	32	62	44.3	100	0	0	0	0
Zn	46.2	86.8	71.2	100	0	0	0	0
Mn	368	801	513.9	100	0	0	0	0
Co	11	19.1	16	100	0	0	0	0
Se	0.066	0.03	0.09	100	0	0	0	0
Be	2.2	2.8	2.4	100	0	0	0	0

**Table 5:** Ecological risk index for heavy metals in the background area soil.

Heavy Metal	Min	Max	Mean	Slight (%)	Moderate (%)	Intense (%)	Very Strong (%)	Extremely Strong (%)
As	7	16.8	11.1	100	0	0	0	0
Cd	0.1	0.5	0.2	50	50	0	0	0
Cu	14	38	26.9	100	0	0	0	0
Pb	7.7	29.5	14	100	0	0	0	0
Hg	0	0.5	0.1	25	50	25	0	0
Ni	32	62	44.3	100	0	0	0	0
Zn	46.2	86.8	71.2	100	0	0	0	0
Mn	368	801	513.9	100	0	0	0	0
Co	11	19.1	16	100	0	0	0	0
Se	0	0.1	0.1	100	0	0	0	0
Be	2.2	2.8	2.4	100	0	0	0	0

**Table 6:** Statistical analysis of PAH concentrations in soil.

PAHs	Ring Content	Max (mg/kg)	Mean (mg/kg)	Standard Deviation	Limit of Detection (μg/kg)	CV	Eco-SSLs (μg/kg)
Baa	4	0.015	0.002	0.005	4	2.072	15
Bap	5	0.046	0.004	0.013	5	3.464	1.5
Bbf	5	0.023	0.002	0.007	5	3.464	15
Bkf	5	0.008	0.001	0.002	5	3.464	151
Chr	4	0.025	0.006	0.009	3	1.601	1293
Daha	5	0.118	0.015	0.035	5	2.382	0.55
NaP	2	0.022	0.005	0.008	3	1.85	70
Acy	3	0.039	0.006	0.014	5	2.349	45,000
Flu	3	0.141	0.02	0.039	3	1.934	2000
Phe	3	0.051	0.005	0.015	5	2.967	40
Ant	3	0.183	0.043	0.056	4	1.292	2000
Fla	4	0.052	0.012	0.018	5	1.494	2000
Pyr	4	0.646	0.162	0.203	3	1.252	1700

**Table 7:** ILCRs and CR for adults and children under different exposure routes.

Group	Statistic	ILCRs_cib_	ILCRS_skin_	ILCRs_bre_	CR
Adult	Max	9.85 × 10^−7^	1.53 × 10^−9^	1.62 × 10^−7^	1.15 × 10^−6^
	Min	3.03 × 10^−10^	4.71 × 10^−13^	4.98 × 10^−11^	3.53 × 10^−10^
	Mean	4.93 × 10^−7^	7.66 × 10^−10^	8.11 × 10^−8^	5.74 × 10^−7^
Child	Max	1.56 × 10^−6^	2.43 × 10^−9^	2.57 × 10^−7^	1.82 × 10^−6^
	Min	4.81 × 10^−10^	7.47 × 10^−13^	7.91 × 10^−11^	5.60 × 10^−10^
	Mean	2.70 × 10^−7^	4.20 × 10^−10^	4.44 × 10^−8^	3.15 × 10^−7^

**Table 8:** Concentration of heavy metals in groundwater and water quality standards.

Heavy Metals	S01 (μg/L)	S00 (μg/L)	Limit of Detection (μg/L)	Class IV Water Quality Standards
As	3.8	-	0.3	≤0.05 mg/L
Cd	-	-	0.1	≤0.01 mg/L
Pb	-	-	1	≤0.1 mg/L
Cu	-	-	9	≤1.5 mg/L
Hg	-	-	0.04	≤0.002 mg/L
Se	-	-	0.4	5.5 ≤ pH < 6.5, 8.5 < pH ≤ 9.0
Be	-	-	0.2	≤0.06 mg/L
Ni	-	-	6	≤0.10 mg/L
Zn	1.00	2.00	1	5.00 mg/L
Mn	105.00	54.00	0.5	1.50 mg/L

Note: “-” indicates that the concentration of pollutants was below the detection limit.

## Data Availability

The original data presented in the study are included in the article; further inquiries can be directed to the corresponding author.
